# Asthma and all-cause mortality in children and young adults: a population-based study

**DOI:** 10.1136/thoraxjnl-2020-214655

**Published:** 2020-09-22

**Authors:** Emma Caffrey Osvald, Hannah Bower, Cecilia Lundholm, Henrik Larsson, Bronwyn K Brew, Catarina Almqvist

**Affiliations:** 1 Paediatric Allergy and Pulmonology Unit, Astrid Lindgren Children's Hospital, Stockholm, Sweden; 2 Department of Medical Epidemiology and Biostatistics, Karolinska Institutet, Stockholm, Sweden; 3 School of Medical Sciences, Örebro Universitet - Campus USÖ, Örebro, Sweden

**Keywords:** paediatric asthma, asthma epidemiology, asthma

## Abstract

**Background:**

Studies suggest an increased all-cause mortality among adults with asthma. We aimed to study the relationship between asthma in children and young adults and all-cause mortality, and investigate differences in mortality rate by also having a life-limiting condition (LLC) or by parental socioeconomic status (SES).

**Methods:**

Included in this register-based study are 2 775 430 individuals born in Sweden between January 1986 and December 2012. We identified asthma cases using the National Patient Register (NPR) and the Prescribed Drug Register. Those with LLC were identified using the NPR. Parental SES at birth (income and education) was retrieved from Statistics Sweden. We estimated the association between asthma and all-cause mortality using a Cox proportional hazards regression model. Effect modification by LLC or parental SES was studied using interaction terms in the adjusted model.

**Results:**

The adjusted hazard rate (adjHR) for all-cause mortality in asthma cases versus non-asthma cases was 1.46 (95% CI 1.33 to 1.62). The highest increased rate appeared to be for those aged 5–15 years. In persons with asthma and without LLC, the adjHR remained increased at 1.33 (95% CI 1.18 to 1.50), but differed (p=0.002) from those with asthma and LLC, with an adjHR of 1.87 (95% CI 1.57 to 2.22). Parental SES did not alter the association (income, p=0.55; education, p=0.83).

**Conclusion:**

This study shows that asthma is associated with an increased mortality in children and young adults regardless of LLC or parental SES. Further research is warranted to investigate the possible mechanisms for this association.

**Key messages**

**What is the key question?**

- Is asthma associated with increased all-cause mortality rate in children and young adults and does it differ for those with or without life-limiting conditions or by parental socioeconomic status at birth?

**What is the bottom line?**

- The results suggest that asthma increases all-cause mortality also in children and young adults, and while having a life-limiting condition appears to be an important effect modifier for the association between asthma and all-cause mortality, parental socioeconomic status did not alter the association.

**Why read on?**

- To our knowledge, this is the largest population study of children and young adults on this topic, and this study contributes to the further understanding of the association between asthma and all-cause mortality.

## Introduction

All-cause mortality and asthma-specific mortality in children and young adults have reduced in the past decades in Europe and other high-income countries globally.^1 2^ In Sweden, both child mortality and asthma-specific mortality are low.^3 4^ As in other Northern European countries, the leading causes of death in Sweden in childhood and adolescence are road injuries, congenital birth defects and neoplasms.^1 5^ Morbidity among children and young adults in Western Europe is increasingly dominated by non-communicable disease, where asthma is a significant contributor.^5^

The worldwide reduction in asthma-specific mortality is attributed to widespread use of inhaled corticosteroids (ICS) after its introduction in the 1980s.^2 6^ Only 37 deaths from asthma in Sweden were reported for persons aged 1–34 years from 1994 to 2003.^4^ A study on asthma-specific mortality using data from WHO Mortality Database showed that age-standardised mortality in Sweden for persons aged 5–34 years in Sweden has continued to fall.^6^

Although asthma-specific mortality has reduced, there are studies which suggest that having asthma increases the risk of all-cause mortality in adults.^7 8^ A cohort study in Finland found that persons over 30 years of age with asthma were at higher risk of all-cause mortality.^7^ Furthermore it has been shown that adults with asthma are more likely to die from chronic conditions than individuals without asthma.^8^ Results from a recent cohort study from Scotland demonstrate there is also an increased all-cause mortality among children with asthma.^9^ However, the relationship between asthma and all-cause mortality in children and young adults is not as extensively studied and is clearly not fully understood.

Asthma is a chronic condition characterised by symptoms such as wheezing and coughing.^10^ The prevalence among Swedish children is around 10%.^11^ Asthma is known to be comorbid with diseases such as allergic rhinitis, attention deficit hyperactivity disorder and obesity,^10 12–14^ and it has also been shown that persons with asthma more commonly have other chronic conditions than those without asthma.^15^ Chronic conditions also comprise life-limiting conditions, defined as conditions ‘without a reasonable hope of cure’.^16^ Studies from the UK show a rising prevalence of children and young adults with a life-limiting condition, and chronic conditions are increasingly found among children who die.^16 17^

It is well established that disadvantaged socioeconomic status (SES) is associated with an increase in childhood mortality.^18^ Income, education and occupation are often used as measures of SES.^19^ Low SES and factors related to low SES have been shown to increase all-cause mortality in young children, older age groups and asthma-specific mortality.^3 7^ In Sweden, deaths due to asthma have been linked with poor compliance with asthma treatment and substance misuse, both of which are associated with disadvantaged SES.^4^ Low parental SES has also been implicated in the development of asthma in childhood, an increased prevalence of severe asthma and a decreased amount of dispensed controller medication.^19–21^

It is of interest to healthcare providers and policy makers to understand the relationship between asthma, all-cause mortality, life-limiting conditions and parental SES. Hence, the aim of this study was to investigate the association between asthma and all-cause mortality in children and young adults aged 1–25 years and explore if this effect was different for those with or without life-limiting conditions or by parental SES at birth. The hypothesis was that asthma increases all-cause mortality in children and young adults and that a life-limiting condition and parental SES alter this effect.

## Methods

### Study design and population

This was a register-based cohort study of individuals born in Sweden. Information was gathered from four registers held by the Swedish National Board of Health and Welfare: the Medical Birth Register (MBR), the National Patient Register (NPR), the Prescribed Drug Register (PDR) and the Swedish Cause of Death Register. Information from four registers held by Statistics Sweden was also used. These were the Multi-Generation Register (MGR), the Total Population Register (TPR), the Income and Taxation register (IOT), and the Longitudinal Integration Database for Health Insurance and Labour Market Studies (LISA by Swedish acronym). All data from the study individuals and their parents were linked using the personal identification number, a unique identifier held by every person residing in Sweden (online supplemental figure S1).

10.1136/thoraxjnl-2020-214655.supp1 Supplementary data

The study population consists of all individuals born in Sweden between 1 January 1986 and 30 December 2012 (n=2 795 154) identified from the MBR (which reports 99% of all births since 1973).^22^ Those who died (n=10 757, 0.4%) or emigrated (n=8967, 0.3%) during their first year of life were excluded, resulting in a cohort of 2 775 430 study individuals. Mothers and fathers were linked to the study individuals through the MGR.

### Exposure

Asthma cases were identified using data from two registers, the NPR and the PDR, and we based on doctors’ diagnosed asthma and/or on dispensed asthma medications from a doctor’s prescription according to a previously published validation study.^11^ Asthma was defined up to 1 July 2005 using the NPR only, which covers all inpatient hospital care from 1987 and 75% of outpatient visits from 2001. From 1 July 2005 asthma was defined by an algorithm used in a previous validation study, using the NPR as well as the PDR, which covers all prescribed drugs dispensed at pharmacies in Sweden.^11 22^ From the NPR, asthma cases and incidence dates were defined as the first hospital admission or outpatient visit, with a primary or secondary diagnosis of asthma identified using the corresponding code from the International Classification of Diseases (ICD). For ICD-9 code 493 was used and for ICD-10 codes J45 and J46 were used. From the PDR, an asthma case was identified by the dispensed asthma medications, ICS, β-agonists, combination products or leukotriene receptor antagonists, using the following corresponding ATC codes (Anatomic Therapeutic Chemical classification codes): R03BA, R03AC, R03AK and R03DC, respectively. A record of at least two dispensed prescriptions of asthma medications was required to be identified as an asthma case using the PDR. Although it could lead to underestimation of the association, in order to avoid immortal time bias the incident date was determined by the date of the second dispensed prescription. In instances where an individual had an incident date recorded in both the NPR and the PDR, the earliest date was used.

### Outcome

The cohort was linked to the Swedish Cause of Death Register, a register with a high level of completeness and where all deaths from 1961 are recorded.^23^ Data collected included the date of death and cause of death. The outcome, all-cause mortality, was defined as any cause of death which occurred between the study individual’s 1st and 25th birthday. The cause of death was identified by the corresponding ICD code and was used for descriptive purposes only.

### Covariates

The covariates were chosen using a directed acyclic graph (online supplemental figure S2).^24^ From the MBR we obtained data relating to the study individuals’ characteristics: sex, prematurity, if they were born small for gestational age (SGA) and calendar year of birth. From the NPR we identified those with a life-limiting condition, using the ICD-10 code and the corresponding ICD-9 code, based on the definition used by Fraser *et al* 16 (online supplemental table S1), which includes neurological conditions, congenital and chromosomal abnormalities. The earliest date of diagnosis was used in instances where an individual had more than one life-limiting condition.

Covariates relating to family characteristics, maternal age at delivery, smoking during first pregnancy trimester and maternal country of birth were retrieved from the MBR and the TPR.

Data on parental SES (income and education) were obtained using the IOT register and the LISA database. Both LISA and IOT are updated annually and these registers record data on individuals 16 years or older living in Sweden. The IOT register contains information regarding income only; it was established in 1968. In 1990 LISA was established and IOT was integrated into this database, which also contains information on education.^25^

Parental income was defined as the highest parental disposable income in the year before the birth of the study individual, divided into quintiles. Parental education was defined as the highest attained education between each parent in the year of the birth of the study individual. Parental education data for study individuals born before the LISA database started (1986–1989) were defined according to data from LISA in 1990. Parental education was categorised as compulsory school (≤9 years), high school (10–12 years) or college/further education (>12 years).

### Statistical analysis

The study individuals were followed from their first birthday until end of follow-up, which was defined as their 25th birthday, date of death, emigration date or 31 December 2013, whichever occurred first.

Crude mortality rates per 100 000 person years with 95% CI by asthma exposure were first calculated, assuming a Poisson distribution.

The Cox proportional hazards model was then used to estimate HR with 95% CI, comparing all-cause mortality in those with and without asthma. Asthma was treated as time-varying exposure in all analyses, whereby an individual contributed towards ‘no asthma’ time-at-risk before being identified as an asthma case and towards ‘asthma’ time-at-risk if and from the time they were identified as an asthma case. Attained age was the underlying timescale used for survival analyses.

Both an unadjusted model and a model adjusted for covariates were fitted. These covariates were sex, prematurity, SGA, life-limiting condition, calendar year of birth, maternal age at delivery, maternal smoking during pregnancy, maternal birth country and parental SES at birth. Life-limiting condition was modelled as a time-varying variable.

We estimated the time-varying effects (HRs) of the exposure in a smooth way by fitting flexible parametric models, with restricted cubic splines (5 df) to model the log baseline cumulative hazard function and 3 df to model the interaction between age and the exposure variable, where asthma and life-limiting conditions were time-varying variables, and the model was adjusted for all the covariates.^26^

Effect modification by life-limiting conditions or parental SES was tested, using Wald test, by introducing an interaction term in the adjusted Cox proportional hazard model between asthma and (1) life-limiting conditions and (2) parental SES at birth.

From 2001 outpatient specialist visits are also available in the NPR. Hence data available from this year are thought to be more complete and could decrease the chance of misclassification bias of the asthma variable. In order to study the effect of this, a subgroup analysis was conducted on those born from 2000. They were studied by similar methods until 31 December 2013.

All statistical analyses were conducted using STATA V.15.1.

## Results

In total 261 322 asthma cases were identified, resulting in a cumulative incidence of asthma of 9.4%. Table 1 shows the characteristics of the study cohort and their family. The mean age of asthma onset was 8.1 years (95% CI 8.0 to 8.1). Asthma cases were more likely to be male, born premature or have been exposed to maternal smoking in pregnancy. Of those with asthma, 6480 individuals (cumulative incidence 2.5%) had a life-limiting condition, and of those without asthma 28 798 individuals (cumulative incidence 1.1%) had a life-limiting condition.

**Table 1:** Background characteristics related to asthma in a Swedish cohort of 2 775 430 individuals

	No asthma	Asthma
n (%)	2 514 108 (90.6)	261 322 (9.4)
Study individuals’ characteristics		
Sex		
Male	1 277 846 (50.9)	147 383 (56.4)
Female	1 236 255 (49.2)	113 939 (43.6)
Prematurity		
Yes (≤37 weeks)	144 043 (5.7)	22 243 (8.5)
No (>37 weeks)	2 382 811 (94.3)	239 079 (91.5)
Small for gestational age		
Yes	56 886 (2.2)	7145 (2.7)
No	2 382 811 (94.8)	245 453 (94.0)
Missing	74 411 (3.0)	8724 (3.3)
Life-limiting condition		
Yes	28 798 (1.1)	6480 (2.5)
No	2 485 310 (98.9)	254 842 (97.5)
Family characteristics		
Maternal age at delivery, years		
<20	52 249 (2.1)	5444 (2.1)
≥20 to <25	420 197 (16.7)	45 615 (17.4)
≥25 to <30	840 276 (33.4)	90 611 (34.6)
≥30 to <35	779 249 (31.0)	79 073 (30.3)
≥35 to <40	349 137 (13.8)	33 960 (13.0)
≥40	71 630 (2.9)	6486 (2.5)
Missing	1370 (0.1)	133 (0.1)
Maternal smoking during pregnancy		
Yes	356 193 (14.2)	39 347 (15.1)
No	2 016 479 (80.2)	206 644 (79.1)
Missing	141 436 (5.6)	15 331 (5.8)
Maternal country of birth		
Sweden	2 066 737 (82.2)	225 482 (86.3)
Denmark, Norway, Finland, Iceland	69 516 (2.8)	6600 (2.5)
Other	374 597 (14.9)	29 214 (11.2)
Missing	3258 (0.1)	26 (<0.1)
Parental income		
Lowest	486 986 (19.4)	46 634 (17.9)
Lower middle	481 091 (19.1)	51 179 (19.6)
Middle	480 864 (19.1)	51 918 (19.9)
Upper middle	480 520 (19.1)	51 276 (19.6)
Highest	483 183 (19.2)	48 684 (18.6)
Missing	101 464 (4.0)	11 631 (4.5)
Parental education		
Compulsory school	161 801 (6.4)	15 364 (5.9)
High school	1 132 088 (45.1)	125 046 (47.8)
College or further education	1 101 778 (43.8)	108 189 (41.4)
Missing	118 441 (4.7)	12 723 (4.9)

The table compares those who received a diagnosis of asthma during follow-up with those who did not receive a diagnosis of asthma during follow-up.

Data on parental SES were available for 95.2% of the cohort. Of those with asthma, 17.9% of parents were in the lowest income quintile compared with those without asthma, where 19.4% of parents were in the lowest quintile. The distribution of parental education for the asthma group showed 5.9% had only compulsory schooling compared with the non-asthma group, where 6.4% had only compulsory schooling. Patterns of missing data of the variables prematurity, SGA and maternal smoking in pregnancy were studied across the parental SES variables. We found no increase in missing data among these groups; therefore, this should not cause bias in the analysis (data not supplied).

### Outcome data

The mean follow-up time was 13.7 years (7.8 SD). There were 6592 individuals who died between 1 and 25 years of age, which is 0.2% of the cohort. The crude mortality rate was 18.1 (95% CI 17.6 to 18.5) per 100 000 person years. The unadjusted all-cause mortality rate for asthma cases was 32.1 (95% CI 29.5 to 34.9) per 100 000 person years, which was greater than for non-asthma cases at 17.4 (95% CI 17.0 to 17.8) per 100 000 person years (table 2).

**Table 2:** Number of deaths and mortality rates by asthma exposure in a Swedish cohort of 2 775 430 individuals

	All	No asthma	Asthma
n	6592	6044	548
Person years at risk	364.2	347.7	17.1
Mean follow-up time, years	13.7	13.7	14.6
Crude mortality rate (per 100 000 person years) (95% CI)	18.1 (17.6 to 18.5)	17.4 (17.0 to 17.8)	32.1 (29.5 to 34.9)

Among those who died, 548 (8.3%) individuals had asthma and 970 (14.7%) individuals had a life-limiting condition. In total there were only 10 deaths where asthma was the leading cause of death (0.15% of all deaths). Of the study individuals who died during follow-up, 24.8% had parents in the lowest income quintile at birth compared with those who did not die during follow-up, where 19.2% had parents in the lowest income quintile at birth. The distribution of parental education at birth among those who died showed that 10.3% of the individuals had parents with only compulsory schooling, compared with those study individuals who did not die during follow-up, where 6.4% had parents with only compulsory schooling (table 3 and online supplemental table S2).

**Table 3:** Characteristics comparing individuals who died or survived the follow-up period

	Died	Survived
n	6592	2 768 838
Study individuals’ characteristics, n (%)		
Asthma		
Yes	548 (8.3)	260 774 (9.4)
No	6044 (91.7)	2 508 064 (90.6)
Sex		
Male	4079 (61.9)	1 421 150 (51.3)
Female	2513 (38.1)	1 347 681 (48.7)
Prematurity		
Yes (≤37 weeks)	715 (10.9)	165 571 (6.0)
No (>37 weeks)	5877 (89.1)	2 603 267 (94.0)
Small for gestational age		
Yes	381 (6.2)	63 650 (2.3)
No	5966 (91.0)	2 622 298 (94.7)
Missing	258 (3.8)	82 890 (3.0)
Life-limiting condition		
Yes	970 (14.7)	34 308 (1.2)
No	5622 (85.3)	2 734 530 (98.8)
Family characteristics		
Maternal age at delivery, years		
<20	253 (3.8)	57 440 (2.0)
≥20 to <25	1539 (23.4)	464 273 (16.8)
≥25 to <30	2235 (33.9)	928 652 (33.5)
≥30 to <35	1674 (25.4)	856 648 (30.9)
≥35 to <40	711 (10.8)	382 386 (13.8)
≥40	179 (2.7)	77 937 (2.8)
Missing	1 (<0.1)	1502 (<0.1)
Maternal smoking during pregnancy		
Yes	1669 (25.3)	393 871 (14.2)
No	4456 (67.6)	2 218 667 (80.1)
Missing	467 (7.1)	156 300 (5.6)
Maternal country of birth		
Sweden	5517 (83.7)	2 286 702 (82.6)
Denmark, Norway, Finland, Iceland	294 (4.5)	75 822 (2.7)
Other	780 (11.8)	403 031 (14.6)
Missing	1 (<0.1)	3283 (0.12)
Parental income		
Lowest	1637 (24.8)	531 983 (19.2)
Lower middle	1294 (19.6)	530 976 (19.2)
Middle	1218 (18.4)	531 564 (19.2)
Upper middle	1122 (17.0)	530 674 (19.2)
Highest	1093 (16.6)	530 774 (19.2)
Missing	228 (3.6)	112 867 (4.08)
Parental education		
Compulsory school	680 (10.3)	176 485 (6.4)
High school	3659 (55.5)	1 253 475 (45.3)
College or further education	1987 (30.1)	1 207 980 (43.6)
Missing	266 (4.1)	130 898 (4.7)

### Main results

**Figure 1:**
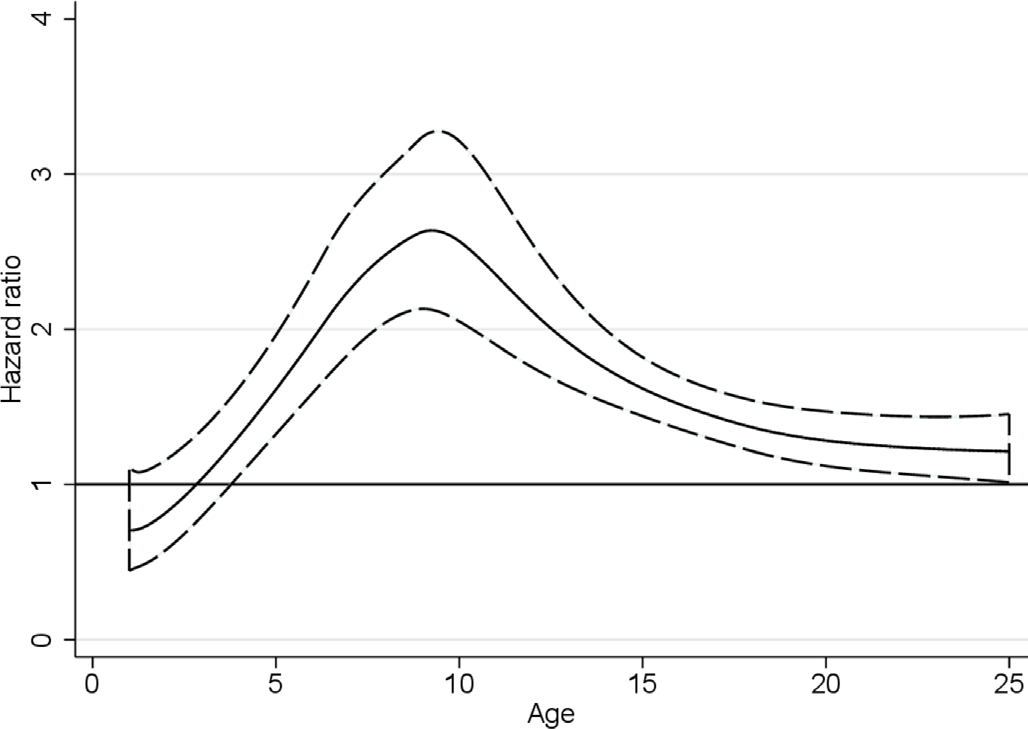
HR with 95% CI comparing all-cause mortality in Swedish individuals aged between 1 and 25 years of age with asthma and those without asthma. HR estimated from the adjusted flexible parametric model.

From the Cox proportional hazard model, the resulting HR for all-cause mortality was 1.68 (95% CI 1.54 to 1.83) by comparing those with asthma with those without asthma. Adjusting for covariates altered the HR to 1.46 (95% CI 1.33 to 1.62). The HR for all-cause mortality varied by age as estimated from the adjusted flexible parametric survival model allowing for a time-varying effect. The largest HR was seen for deaths between 5 and 15 years of age (figure 1).

**Figure 2:**
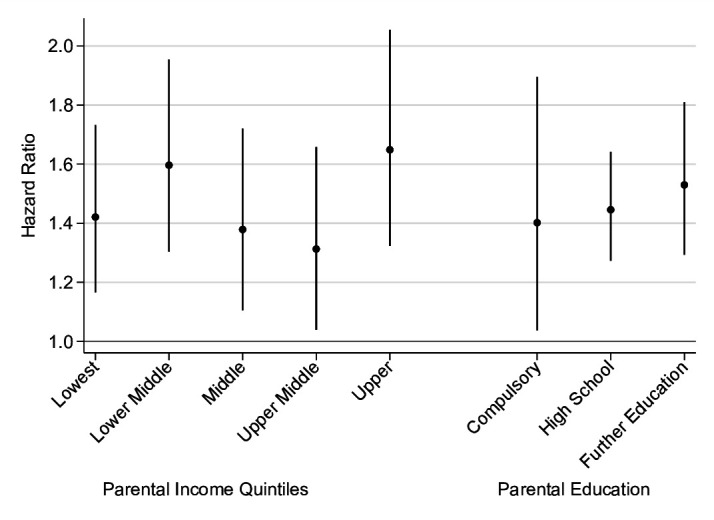
Adjusted HR with 95% CI estimated from Cox proportional hazards models of all-cause mortality comparing individuals with asthma and those without asthma within each parental socioeconomic group.

In persons with asthma and with and without a life-limiting condition, the adjusted hazard rate (adjHR) for all-cause mortality differed (p=0.002), demonstrating a significant interaction. Those with asthma and a life-limiting condition had an adjHR of 1.87 (95% CI 1.57 to 2.22), whereas in those with asthma and without a life-limiting condition the all-cause mortality rate remained increased but was lower in those with a life-limiting condition, with an adjHR of 1.33 (95% CI 1.18 to 1.50). There was no significant interaction for measures of parental SES (income, p=0.55; education, p=0.83). The adjHRs are further depicted in figure 2 (see also online supplemental table S3), which compares the HR within each parental socioeconomic group.

### Subgroup analysis

The subgroup analysis of 1 312 844 individuals born from 2000 included 121 959 asthma cases (cumulative incidence of 9.2%). The adjusted Cox regression model showed an HR of 1.79 (95% CI 1.39 to 2.08).

## Discussion

This population-based cohort study of 2 775 430 individuals supports the hypothesis that the all-cause mortality rate in children and young adults is higher in those with asthma compared with those without asthma. We expand these findings by showing that having a life-limiting condition altered the rate between the asthma and non-asthma groups, whereas parental SES at birth did not. Few studies have investigated the relationship between asthma and all-cause mortality in younger people. To our knowledge, this is the largest population study of children and young adults on this topic, and this study adds to our understanding of the research field.

Studies on adult populations have demonstrated that asthma increases all-cause mortality,^7 8^ and our study further demonstrates that this association is seen in children and young adults. The recent study from Fleming *et al* 9 has shown an increased all-cause mortality in children with asthma and our subgroup analysis of children aged 1–14 years old shows a similar HR to this study. Furthermore we have demonstrated that the hazard rate for the association between asthma and all-cause mortality changes with age, with the highest rate ratio in children aged 5–15 years old. This observation has previously not been reported and warrants further exploration.

Asthma is known to be comorbid with other chronic conditions, including life-limiting conditions.^15 27^ Our results, perhaps unsurprisingly, suggest that for children and young adults with a life-limiting condition the association between asthma and all-cause mortality is higher than for those without a life-limiting condition; however, what is more interesting to note is that the association between asthma and all-cause mortality remains even for those not identified as having a life-limiting condition. Studies investigating adults with asthma have shown an increased all-cause mortality among adults with other underlying disease,^28^ and others propose that airflow limitation (FEV_1_) is an important component contributing to mortality.^29 30^ Perhaps the relationship between asthma and all-cause mortality is related to increased susceptibility to factors associated with asthma comorbidities, other chronic disease and/or reduced lung function, which could be explored in future research. There is overwhelming evidence that SES in childhood impacts not only health outcomes and mortality in childhood but also adulthood.^31 32^ Evidence of this can be seen in this study, as study individuals who died had a higher proportion of parents from the lowest socioeconomic groups at birth (lowest income quintile and compulsory schooling only) compared with those who survived. Studies in adults with asthma have demonstrated an increased risk of both all-cause mortality and asthma-specific mortality among those from the lowest SES group compared with the highest SES group.^8^ Although we could not demonstrate a significant difference in the association of asthma and all-cause mortality between parental SES groups, parental SES is an important confounder in the relationship between asthma and all-cause mortality.^18^

The Swedish healthcare system is often cited as a system with an established healthcare structure of community and hospital services with the linkage of several patient medical record sources. This creates the right environment for the management of persons with chronic conditions.^5 18^ Healthcare in Sweden is universal, access to emergency care is uniform, and prescriptions for medications during the study period were subsidised. Despite this, the results of this study demonstrate an increased mortality rate among children and young adults with asthma, supporting the argument for the integrated management of chronic conditions in children and young adults and in particular in the treatment of asthma.

### Strengths and limitations

A strength of this study is the large population base, which minimises selection bias and increases generalisability. The variables retrieved from the NPR and the Swedish Cause of Death Register have been shown to have a high positive predictive value, and similarly data pertaining to parental SES at birth were derived from registers which have been validated.^23 25 33^

There are some limitations. First, the study population included only individuals born in Sweden. Although this creates a selection bias, as it does not include individuals who immigrate to Sweden, it minimises misclassification bias with regard to the asthma variable and data pertaining to the pregnancy, perinatal outcomes and parental SES, which are not available for persons who are not born in Sweden. Second, the asthma variable was created largely from hospital data and hence we may have found more serious asthma cases and missed milder cases. In order to overcome this bias and capture more individuals with asthma who are managed in primary care, we have used data from the Swedish PDR, which started in 2005. Asthma cases were defined in accordance with a validation study which found a high positive predictive value for asthma defined using both the NPR and/or the PDR.^11^ Furthermore the subgroup analysis of those born from 2000 indicates that potential bias from a misclassification of the asthma variable probably has limited impact on the findings, as we demonstrate that the increased all-cause mortality persists for those with asthma. Third, we did not have access in the NPR to all the life-limiting conditions defined by Fraser *et al*,^16^ which might result in a misclassification bias and might affect our estimates in our adjusted model. Finally, we had access to data on maternal smoking during pregnancy but no other information on exposure to tobacco smoke or individual smoking habits. Smoking has been demonstrated as a significant factor which increases all-cause mortality in adults with asthma and differs within socioeconomic groups.^7^ In 2007, 14% of the Swedish population smoked and around 7% of the population reported daily environmental smoke exposure.^34^ Therefore, the bias from not having smoking data may be minimal; however, we cannot rule smoke exposure out altogether. Future research on asthma and all-cause mortality in children and young adults should further explore the effect of smoking and smoke exposure on this association.

## Conclusion and implications

To our knowledge this is the first study specifically examining the relationship between asthma and all-cause mortality in children and young adults. While this study shows that asthma is associated with an increased rate of death in children and young adults, the asthma-specific mortality is still relatively low. The HR comparing the mortality rate in the asthma and non-asthma groups was increased by also having a life-limiting condition, whereas parental SES at birth did not alter the association between asthma and all-cause mortality. The study provides further evidence that those with asthma have an increased all-cause mortality, which has important clinical implications and strengthens the argument for continued research in this field.
