# Supplementary material for: Asthma and all-cause mortality in children and young adults: a population-based study
Source: Thorax. 2020 Sep 22;75(12):1040–6. doi: 10.1136/thoraxjnl-2020-214655 (PMC7677462; doi:10.1136/thoraxjnl-2020-214655)
Supplement: Supplementary data [file thoraxjnl-2020-214655supp001.pdf]

## Supplement

## Figure Legend:

Figure S 1: Data sources

Figure S 2
Directed Acyclic Graph depicting the exposure (asthma), the outcome (death 1-25 years of age) and the potential confounders: sex, prematurity, SGA, calendar year of birth, life-limiting condition, maternal age at delivery, smoking during pregnancy, birth country, and parental SES.



Caffrey Osvald E, *et al. Thorax* 2020; 75:1040–1046. doi: 10.1136/thoraxjnl-2020-214655

Figure S1:

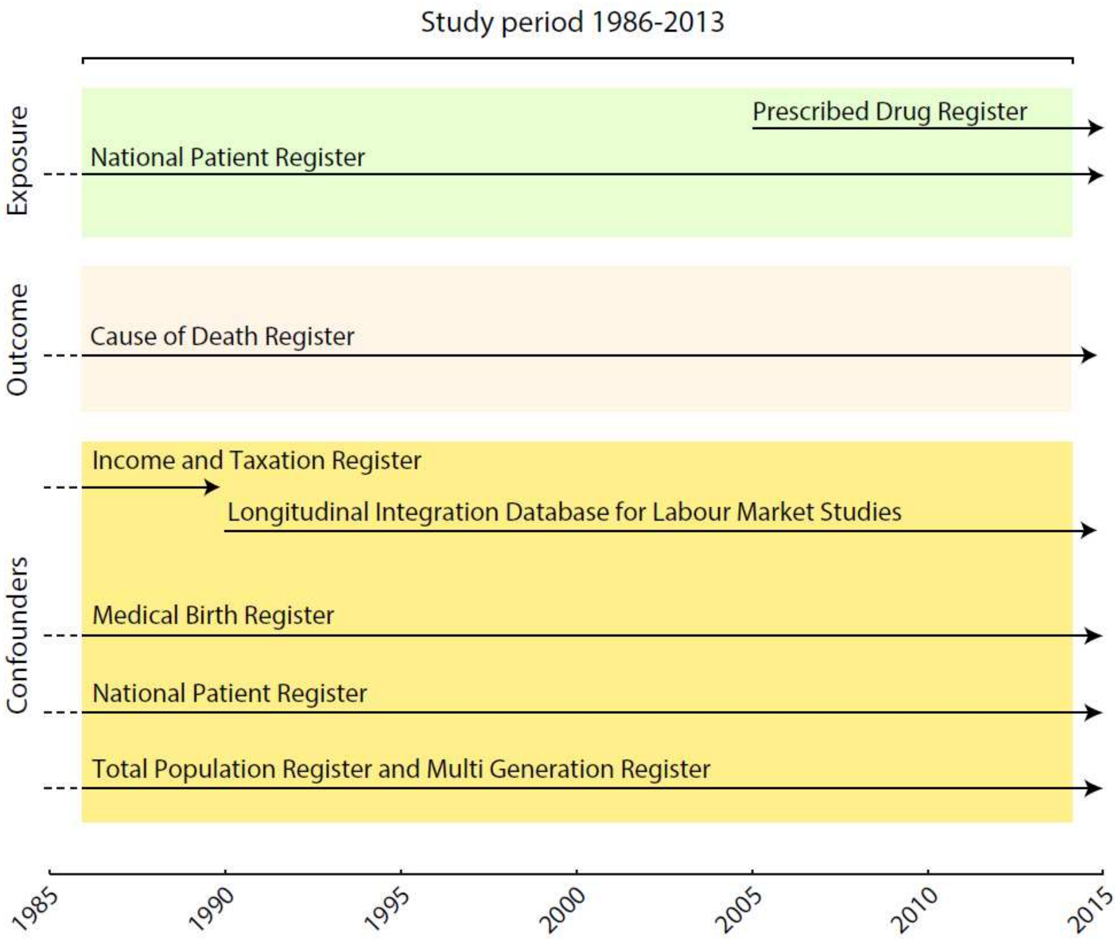




Caffrey Osvald E, *et al. Thorax* 2020; 75:1040–1046. doi: 10.1136/thoraxjnl-2020-214655

Figure S2:

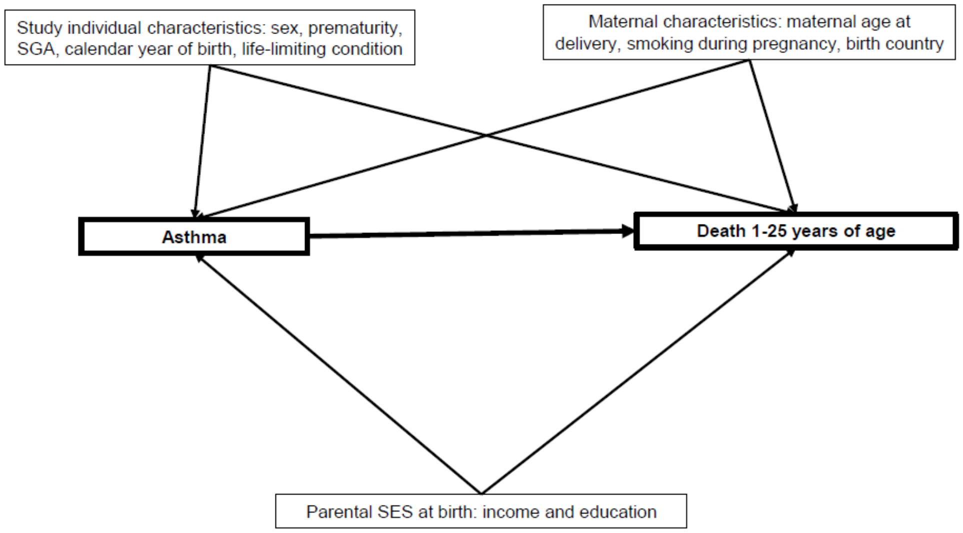




Caffrey Osvald E, *et al. Thorax* 2020; 75:1040–1046. doi: 10.1136/thoraxjnl-2020-214655

**Table S1: ICD-10 codes used to identify Life-Limiting Conditions in children and young adults**

| **A** | **B** | **D** | **E** | **G** | **G** | **I** | **M** |
|---|---|---|---|---|---|---|---|
| A17 | B20-B24 | D81 | E31.0 | G10 | G40.4 | I21 | M31.3 |
| A81.0 | | D82.1 | E34.8 | G11.1 | G40.5 | I27.0 | M32.1 |
| A81.1 | | D83 | | G11.3 | G60 | I42 | M89.5 |
| | | D89.1 | | G12 | G60.1 | 161 | |
| | | | | G20 | G70.2 | | |
| | | | | G23.0 | G70.9 | | |
| | | | | G23.8 | G71.0 | | |
| | | | | G31.8 | G71.1 | | |
| | | | | G31.9 | G71.2 | | |
| | | | | G35 | G71.3 | | |
| **P** | **Q** | **Q** | **Q** | **Q** | **Q** | **T** | |
| P10.1 | Q00.0 | Q21.9 | Q28.2 | Q74.8 | Q87.1 | T86.0 | |
| P11.2 | Q01 | Q22.0 | Q32.1 | Q75.0 | Q87.8 | T86.2 | |
| P21.0 | Q03.1 | Q22.1 | Q33.6 | Q77.2 | Q91-Q95 | | |
| P28.5 | Q03.9 | Q22.4 | Q34 | Q77.3 | | | |
| P29.0 | Q04.0 | Q22.5 | Q39.6 | Q77.4 | | | |
| P29.3 | Q04.4 | Q22.6 | Q41.0 | Q78.0 | | | |
| P35.0 | Q04.6 | Q23.0 | Q43.7 | Q78.5 | | | |
| P35.8 | Q04.9 | Q23.2 | Q44.2 | Q79.2 | | | |
| P37.1 | Q07.0 | Q23.4 | Q44.5 | Q79.3 | | | |
| P52.5 | Q20.0 | Q23.9 | Q44.7 | Q80.4 | | | |
| P52.9 | Q20.3 | Q25.4 | Q60.1 | Q81 | | | |
| P83.2 | Q20.4 | Q25.6 | Q60.6 | Q82.1 | | | |
| P91.2 | Q20.6 | Q26.2 | Q61,9 | Q85.8 | | | |
| P91.6 | Q20.8 | Q26.4 | Q64.2 | Q86.0 | | | |
| P96.0 | Q21.3 | Q26.8 | Q74.3 | Q87.0 | | | |



Caffrey Osvald E, *et al. Thorax* 2020; 75:1040–1046. doi: 10.1136/thoraxjnl-2020-214655

**Table S2: Causes of death comparing those with and without asthma**

| | All | No Asthma | Asthma |
|---|---|---|---|
| **N (%)** | 6592 | 6044 | 548 |
| **Cause of death** | | | |
| **Injury Poisoning** | 2780 (42.2) | 2637 (43.6) | 143 (26.1) |
| **Neoplasm** | 1113 (16.9) | 1049 (17.4) | 64 (11.7) |
| **Congenital** | 543 (8.2) | 469 (7.8) | 74 (13.5) |
| **Neurological** | 516 (7.8) | 420 (7.0) | 96 (17.5) |
| **Endocrine** | 393 (6.0) | 360 (6.0) | 33 (6.0) |
| **Cardiac** | 303 (4.6) | 273 (4.5) | 30 (5.5) |
| **Respiratory*** | 135 (2.1) | 111 (1.8) | 24 (4.4) |
| **Other **** | 809 (12.3) | 724 (12.0) | 84 (15.3) |

*Includes 10 deaths from asthma **Other: deaths from infections, haematological, psychiatric, gastrointestinal, pregnancy, dermatological and musculoskeletal causes based on ICD code



Caffrey Osvald E, *et al. Thorax* 2020; 75:1040–1046. doi: 10.1136/thoraxjnl-2020-214655

**Table S3: Adjusted hazard ratios with 95% confidence intervals comparing the all-cause mortality in the asthma group to the no asthma group estimated from Cox proportional hazards models, with p-values for test of interaction with asthma exposure.**

| | HR (95% CI) | p-value |
|---|---|---|
| **Life limiting condition** | | 0.002 |
| Yes | 1.87 (1.57-2.22) | |
| No | 1.33 (1.18-1.50) | |
| | | |
| **Parental Income** | | 0.55 |
| Lowest | 1.42 (1.17-1.73) | |
| Lower Middle | 1.60( 1.30-1.95) | |
| Middle | 1.38 (1.11- 1.72) | |
| Upper Middle | 1.31(1.04-1.66) | |
| Highest | 1.65 (1.32-2.05) | |
| | | |
| **Parental Education** | | 0.83 |
| Compulsory school | 1.40 ( 1.04-1.90) | |
| High School | 1.45 ( 1.27-1.64) | |
| College or Further Education | 1.53 ( 1.29-1.81) | |

HR=hazard ratio. Cox proportional hazard regression model adjusted for sex, prematurity, SGA, life-limiting condition, birth year, maternal age at birth, maternal smoking during pregnancy, maternal country of birth, parental disposable income and highest parental education at birth, in an interaction model with life-limiting condition or parental SES.
